# Recognition of Authentic Happy and Sad Facial Expressions in Chinese Elementary School Children: Evidence from Behavioral and Eye-Movement Studies

**DOI:** 10.3390/bs15081099

**Published:** 2025-08-13

**Authors:** Qin Wang, Huifang Xu, Xia Zhou, Wanjala Bakari, Huifang Gao

**Affiliations:** 1 Key Research Base of Humanities and Social Sciences of the Ministry of Education, Academy of Psychology and Behavior, Tianjin Normal University, Tianjin 300387, China; 2 Faculty of Psychology, Tianjin Normal University, Tianjin 300074, China; 3 Tianjin Key Laboratory of Student Mental Health and Intelligence Assessment, Tianjin 300387, China

**Keywords:** facial expressions, authenticity discrimination, gaze patterns, elementary school children

## Abstract

Accurately discerning the authenticity of facial expressions is crucial for inferring others’ psychological states and behavioral intentions, particularly in shaping interpersonal trust dynamics among elementary school children. While existing literature remains inconclusive regarding school-aged children’s capability to differentiate between genuine and posed facial expressions, this study employed happy and sad facial stimuli to systematically evaluate their discrimination accuracy. Parallel to behavioral measures, children’s gaze patterns during authenticity judgments were recorded using eye-tracking technology. Results revealed that participants demonstrated higher accuracy in identifying genuine versus posed happy expressions, whereas discrimination of sad expressions proved more challenging, especially among lower-grade students. Overall, facial expression recognition accuracy exhibited a positive correlation with grade progression, with visual attention predominantly allocated to the Eye-region. Notably, no grade-dependent differences emerged in region-specific gaze preferences. These findings suggest that school-aged children display emotion-specific recognition competencies, while improvements in accuracy operate independently of gaze strategy development.

## 1. Introduction

Facial expressions (the externalization of internal emotional states via coordinated facial muscle movements) exhibit cross-cultural universality in their production and interpretation (Ekman, 1993; Ekman & Friesen, 1971). As primary channels of affective communication, facial expressions enable individuals to evaluate social rewards or threats and adapt behavior accordingly. However, genuine emotions may not always align with observable facial displays (Zloteanu et al., 2021). People often deliberately adjust their facial displays to hide real feelings, such as forcing smiles or pretending to cry (Ekman & Friesen, 1971; Liu & Fang, 2004). Misinterpreting such posed expressions as authentic can trigger inappropriate emotional and behavioral responses (Feng et al., 2020). Therefore, the ability to accurately recognize the authenticity of expressions is vital for social trustworthiness and interpersonal safety (He et al., 2021).

The rapid and accurate detection of facial expression authenticity has garnered considerable scholarly interest. Darwin’s inhibition hypothesis posits that when people actively suppress or mask their genuine emotions, certain facial muscles that are difficult to activate or control autonomously escape voluntary efforts, thereby revealing true feelings (Ekman, 2003). This implies that posed expressions may exhibit detectable inconsistencies. For instance, the Duchenne marker (the concurrent contraction of the zygomatic major and orbicularis oculi muscles) serves as a psychophysiological signature of spontaneous happiness, distinguishing genuine from posed smiles (Ekman et al., 1988, 1990; Miller et al., 2022). Similarly, authentic sadness is characterized by brow furrowing, medial forehead wrinkling, and mouth corner depression (Fu, 2022). Empirical evidence demonstrates that facial expression recognition prioritizes localized facial processing (Y. Song & Hakoda, 2012), with the eyes playing a critical role in decoding emotional cues (Guarnera et al., 2017; Mai et al., 2011; Sui & Ren, 2007). When feigning happiness or sadness, individuals can voluntarily control zygomaticus major contraction and the lip corner elevation/depression. However, simultaneously orchestrating the orbicularis oculi activation and brow movements to generate authentic periorbital affective characteristics proves challenging, as these involuntary responses resist conscious command. Such involuntary muscular activities provide reliable indicators for detecting posed expressions (Ekman & Rosenberg, 1997).

Recognizing genuine facial expressions can be challenging due to the subtlety of the key cues that indicate their authenticity. The Perceptual–Attentional Mechanisms (PAMs) maintain that emotion recognition depends on selectively attending to diagnostically critical cues (e.g., periocular micro-expressions) and elaboratively processing their features to detect configuration discrepancies between genuine and posed expressions in critical regions (Del Giudice & Colle, 2007). Gosselin et al. (2002) employed highly controlled facial stimuli to isolate authenticity markers. Their findings revealed that 6- to 7-year-old children failed to discriminate smile authenticity and could not localize the relevant cues, whereas 9- to 10-year-olds and adults successfully identified genuine smiles when viewing full temporal dynamics (onset–apex–offset sequences), implicating Eye-region changes as critical discriminative cues. The validity of this theoretical framework is evidenced in autism spectrum disorder (ASD) research. Webster et al. (2021) substantiate impaired authenticity discrimination linked to atypical gaze patterns in this population. Critically, Boraston et al. (2008) demonstrated that individuals with ASD exhibit both significantly reduced Eye-region fixation and authenticity discrimination deficits, with ocular inattention mechanistically explaining diminished accuracy. This causal relationship is further supported by intervention studies showing that redirecting gaze to Eye-region cues improves recognition accuracy in ASD (Black et al., 2017). However, counterevidence emerges from studies observing no systematic correlation between gaze patterns and recognition accuracy (Manera et al., 2011; Perron & Roy-Charland, 2013). These findings suggest that successful recognition may depend more fundamentally on the cognitive interpretation of diagnostic cues than on attentional allocation alone.

A robust age-dependent pattern emerges in emotional authenticity discrimination (Scarpazza et al., 2025; Dawel et al., 2015; T. McLellan et al., 2010; T. L. McLellan et al., 2012). Empirical evidence demonstrates that adults exhibit superior ability to differentiate genuine from posed expressions across distinct emotional categories (e.g., happy, fear, and sad). However, the developmental trajectories underlying this capacity remain underexplored, regarding how such skills evolve from childhood to adulthood. Eye-tracking studies reveal distinct gaze patterns between children and adults during facial expression processing, characterized by significant variations in regional attention allocation (Gu & Bai, 2014). This raises critical questions: Do children’s authenticity detection errors stem from divergent gaze strategies? Is developmental improvement associated with the maturation of gaze patterns? Currently, no studies directly characterize children’s gaze distribution across facial regions during authenticity judgments. To address this gap, our study employs eye-tracking technology to investigate developmental trajectories of authenticity detection in elementary school children (Grades 1–6), exploring relationships between regional fixation duration and discrimination accuracy while evaluating the applicability of the PAM framework to childhood. 

Facial expression category constitutes a critical determinant in children’s authenticity discrimination. Empirical studies indicate that young children develop the ability to recognize facial expressions using visual cues at an early age, achieving relative stability after age 5 with proficient recognition of happy and sad (Widen & Russell, 2003). In contrast to basic emotion recognition, discriminating expression authenticity requires higher-order cognitive processing, and current research reveals inconsistent developmental trajectories regarding children’s capacity to identify authenticity across different expressions. For instance, R. Song et al. (2016) demonstrated that 3-year-olds fail to reliably discriminate genuine from posed smiles, whereas 4-year-olds achieve recognition accuracy significantly above chance level. Conversely, Gosselin et al. (2002) reported successful authenticity discrimination only in 9–10-year-olds, with elementary school children aged 6–7 showing no such capability. Dawel et al. (2015) revealed that 8–12-year-olds could identify happy authenticity but not sad authenticity, observing no significant age-related progression during the elementary years. These inconsistencies are potentially attributable to methodological variations in stimulus presentation and task complexity. To systematically clarify developmental trajectories, our study utilizes standardized static facial stimuli and a single-trial presentation paradigm, focusing on happy and sad—two basic emotions crucial for social engagement.

The present study investigated the development of emotion authenticity discrimination in elementary school children using happy and sad expressions as stimuli, examining the relationship between gaze patterns and discrimination performance. Our findings would evaluate the PAM framework’s explanatory validity in childhood development and provide insights for intervention strategies. We constructed the following hypotheses: First, emotional category would modulate discrimination accuracy, with higher accuracy for happy compared to sad expressions. Second, consistent with the Perceptual–Attentional Mechanism (PAM) framework, advancing grade levels would be associated with joint increases in both recognition accuracy and visual attention to the Eye-region. Third, correct authenticity judgments would be characterized by significantly longer Eye-region fixation duration compared to incorrect judgments.

## 2. Materials and Methods

### 2.1. Participants

A priori power analysis using G*Power 3.1 with a power of 0.95 and a medium effect size (f = 0.25) indicated a minimum required sample size of 45. Sixty-four elementary school children (Grades 1–6) were initially recruited. Four participants were excluded due to excessive movement or verbal interference during testing. The final sample (N = 60) comprised three grade-based groups: lower (Grades 1–2, n = 20, Mage = 7.14 ± 0.51 years), middle (Grades 3–4, n = 20, Mage = 9.17 ± 0.71 years), and upper grades (Grades 5–6, n = 20, Mage = 10.76 ± 0.53 years) (Chen et al., 2019; Zhu & Zhao, 2023). The sample included equal gender distribution (30 males and 30 females), absence of psychiatric history, and normal/corrected-to-normal visual acuity. All participants were right-handed. This study was approved by the ethics committee of the investigator’s institution. Participants were required to sign informed consent forms and received compensatory gifts after the experiment.

### 2.2. Research Design

A 3 (Grade: lower, middle, and upper) × 2 (authenticity: genuine and posed) × 2 (expression: happy and sad) mixed factorial design was implemented. Grade was a between-subject factor, while authenticity and expression were within-subject factors. Dependent variables included behavioral measures (accuracy and reaction time) and eye-tracking measures (Fixation duration per Area of interest (AOI): Eye-region, Midface, and Mouth).

### 2.3. Apparatus and Materials

Eye movements were recorded monocularly using an Eyelink Portable Duo system (SR Research, Ottawa, ON, Canada) at a 1k Hz sampling rate. Stimuli were displayed on a 15.6-inch monitor (1920 × 1080 resolution, 60 Hz refresh rate) positioned 50 cm from participants, subtending a 10.28° visual angle (image size: 9 × 7.8 cm).

**Figure 1:**
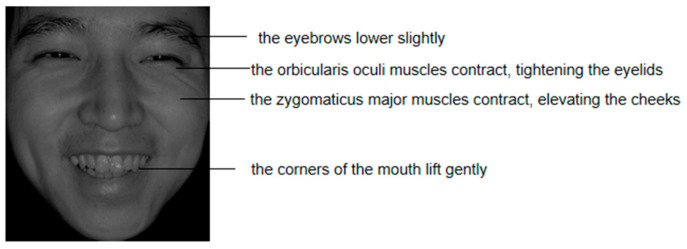
Example authenticity cues in genuine happy facial expressions.

**Figure 2:**
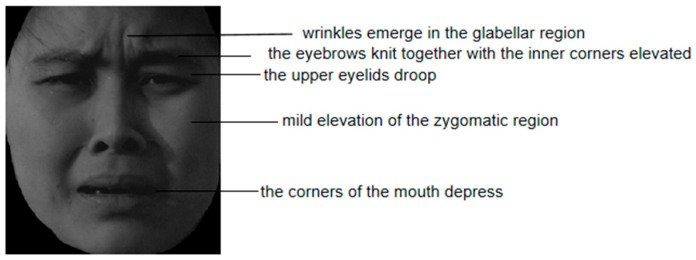
Example authenticity cues in genuine sad facial expressions.

To enhance ecological validity, facial expression stimuli were selected following Dawel et al.’s (2021) framework. We initially selected 30 genuine-happy, 30 posed-happy, 30 genuine-sad, and 30 posed-sad images from the Chinese Affective Face Picture System (CAFPS, Gong et al., 2011) based on established authenticity criteria (Ekman et al., 1990; Fu, 2022). Fifty-four college students rated the genuineness of these facial expressions on a Likert scale (Dawel et al., 2017). For the formal experiment, eight high-genuineness and eight high-posed images per emotion were retained. A repeated-measures ANOVA confirmed significantly higher genuineness ratings for authentic vs. posed stimuli, *F*(1, 30) = 133.33, *p* < 0.001, $\eta_{p}^{2}$ = 0.82. The selected images underwent revalidation of authenticity cues. Genuine expressions consistently exhibited established diagnostic characteristics (genuine happiness as illustrated in Figure 1, and genuine sadness as shown in Figure 2). Conversely, posed expressions failed to concurrently display defining features, particularly lacking subtle periocular micro-activations. AOIs were deliberately designed to encompass authenticity cue regions: Eye-region (orbicularis oculi + corrugator supercilii), Midface (zygomaticus minor + nasal musculature), and Mouth (depressor anguli oris + orbicularis oris).

### 2.4. Procedure

**Figure 3:**
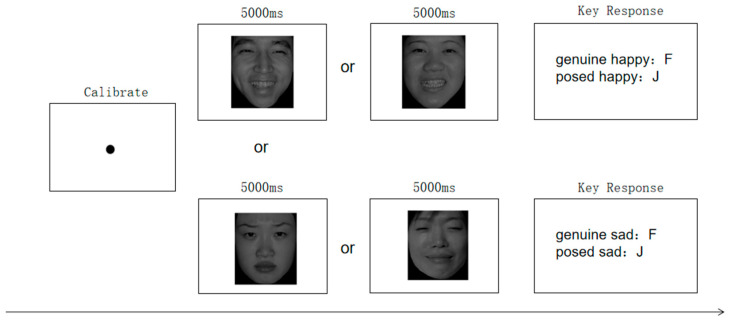
Schematic diagram of the trial sequence.

The experiment began with a brief introduction to the eye-tracking procedure to reduce potential nervousness. Participants were then guided to confirm their understanding of genuine versus posed facial expressions. Following this, the eye tracker was positioned, and the system was calibrated using a 9-point calibration procedure. Calibration accuracy was rigorously verified, requiring an average error of less than 0.5° and no single-point error exceeded 1.5°. To reduce participants’ visual fatigue, the experiment was divided into two blocks separated by a 5-min break, after which a second identical 9-point calibration was performed prior to resuming the task. Each block consisted of 32 trials, with stimulus presentation order fully randomized within blocks. The trial sequence is shown in Figure 3. Participants were required to make a keypress (F or J) response, indicating the genuineness (genuine or posed) of each facial expression. Counterbalancing was not implemented due to task complexity considerations for child participants. To ensure task familiarity, participants completed eight practice trials prior to the formal experiment.

## 3. Results

We employed fixation duration to assess the participant’s fixation time on specific areas of interest. This measure reflects both the depth of cognitive processing in the areas of interest (Wang et al., 2016) and the attentional resource allocation to visual stimuli (Shimojo et al., 2003). Fixation durations shorter than 80 ms were excluded from analysis (Rayner, 2009). Statistical analyses were performed using linear mixed-effects models (LMMs) with the lme4 package in R (version 3.5.2).

### 3.1. Behavioural Results

#### 3.1.1. Accuracy

Means and standard deviations for recognition accuracy (%) of happy and sad facial expressions across grade levels are given in Table 1.

The results demonstrated that middle-grade students exhibited significantly higher accuracy compared to lower-grade students (z = 2.09, *p* = 0.037, 95% CI = [0.02, 0.56]), and upper-grade students also showed significantly higher accuracy than lower-grade students (z = 2.88, *p* = 0.004, 95% CI = [0.13, 0.67]). Significant main effects emerged for expression and authenticity. Happy expressions were recognized more accurately than sad expressions (z = −4.74, *p* < 0.001, 95% CI = [−0.89, −0.39]), and posed expressions showed higher accuracy than genuine expressions (z = 4.04, *p* < 0.001, 95% CI = [0.29, 0.79]). Additionally, an interaction between expression and authenticity was observed. Genuine sad expressions were significantly less accurate than posed sad expressions (z = −5.17, *p* < 0.001, 95% CI = [−1.34, −0.60]). No significant difference emerged between posed and genuine happy expressions. Moreover, a three-way interaction was found among expression, authenticity, and grade level. Students in lower grade demonstrated significantly lower accuracy for genuine happy expressions compared to posed happy expressions (z = −2.16, *p* = 0.031, 95% CI = [−0.97, −0.05]). All grade groups exhibited lower accuracy for genuine sad expressions compared to posed sad expressions (|z|*s* > 3.45, *ps* < 0.001).

#### 3.1.2. Discrimination Index

Based on signal detection theory (SDT), we evaluated children’s facial expression recognition ability through two key measures: hit rate (correct identification of genuine expressions) and the false alarm rate (misclassification of posed expressions). To avoid potential infinite z-scores when probability is 0 or 1, we applied the standard correction method, where both rates were adjusted using the formula (number of hits + 0.5)/(N + 1), with N representing the total number of target expressions presented (genuine expressions for hit rate calculation, posed expressions for false alarm rate calculation). The discrimination index d’ was calculated using the formula d’ = Z_hit_ − Zf_alse alarms_, where Z denotes the inverse of the standard normal cumulative distribution function. This parameter quantifies participants’ ability to discriminate between genuine and posed expressions (Snodgrass & Corwin, 1988; T. McLellan et al., 2010). Following established interpretation criteria, d’ values exceeding 0.5 indicate that participants can accurately detect authenticity of others’ expressions (T. McLellan et al., 2010). Discrimination indices for happy and sad expressions across grade levels are presented in Table 2.

A repeated-measures ANOVA revealed a significant main effect of expression, *F*(1, 61) = 39.767, *p* < 0.001, $\eta_{p}^{2}$ = 0.395. Participants were better at discriminating happy expressions than sad ones. A significant main effect of grade was observed, *F*(2, 61) = 4.057, *p* = 0.022, $\eta_{p}^{2}$ = 0.117. Post-hoc analyses indicated that lower-grade students demonstrated significantly reduced discriminatory capacity relative to upper-grade students (*p* = 0.023, 95% CI = [−0.96, −0.06]). One-sample *t*-tests showed that discrimination indices for authentic happy expressions were significantly greater than 0.5 across all grade levels (|*t*|*s* > 2.91, *ps* < 0.01), while middle and upper grade students also demonstrated significantly above-chance discrimination for authentic sad expressions (|*t*|*s* > 2.65, *ps* < 0.016).

#### 3.1.3. Reaction Time

Means and standard deviations of children’s reaction time in lower, middle, and upper grades under experimental conditions are shown in Table 3.

The linear mixed model analysis indicated a marginal main effect of grade on reaction time (*t* = −1.68, *p* = 0.097, 95% CI = [−776, 59]), suggesting a trend toward faster responses in upper-grade students relative to middle-grade students. Additionally, there was a significant interaction between expression and grade (*t* = 2.85, *p* = 0.004, 95% CI = [100, 541]), indicating that middle- and upper-grade students exhibited significantly shorter reaction time for happy expressions compared to sad expressions (|*t*|*s* > 2.05, *ps* < 0.05).

### 3.2. Eye Movement Results

#### 3.2.1. Fixation Duration (ms) Across Three Areas of Interest (Eye-Region, Midface, and Mouth)

Means and standard deviations of fixation duration (ms) across three AOIs for students across grade levels are presented in Table 4.

Linear mixed model analyses of eye movement data across three grade levels revealed consistent significant three-way interactions between expression, authenticity, and areas of interest in fixation duration measures (|*t*|*s* > 2.11, *ps* < 0.05). Specifically, for genuine happy expressions, fixation duration was significantly longer for the Eye-region compared to both the Midface and Mouth (|*t*|*s* > 7.72, *ps* < 0.001). Similarly, for posed happy expressions, Eye-region fixation duration exceeded both Midface and Mouth (|*t*|*s* > 14.49, *ps* < 0.001). Furthermore, during sad expressions (both genuine and posed), fixation duration remained longest for Eye-region compared to Midface and Mouth (|*t*|*s* > 6.24, *ps* < 0.001), while Mouth fixation duration was significantly shorter than Midface duration (|*t*|*s* > 6.16, *ps* < 0.001).

#### 3.2.2. Gaze Patterns in Correct vs. Incorrect Trials

Means and standard deviations of Eye-region fixation duration (ms) in correct and incorrect trials for students across grade levels are presented in Table 5.

Linear mixed models comparing Eye-region fixation duration between correct and incorrect trials revealed a significant main effect of facial expression (*t* = 3.01, *p* = 0.005, 95% CI = [1726, 2035]). Specifically, fixation duration on the Eye-region was significantly longer when participants judged sad expressions compared to happy expressions. However, the main effect of detection response (Correct vs. Incorrect judgments) was not significant (*p* = 0.575), indicating that attentional allocation patterns do not directly determine single-trial accuracy.

## 4. Discussion

The present study examined elementary school children’s ability to discriminate authenticity in both genuine and posed facial expressions of happy and sad. Results revealed progressive developmental improvement, with upper-grade students demonstrating higher accuracy compared to lower-grade peers, aligning with previously documented age-related gains in authenticity detection (Dawel et al., 2015).

Notably, while lower-grade children showed limited overall accuracy, their discrimination indices for happy expressions surpassed chance levels, indicating an emerging capacity to differentiate genuine smiles, consistent with basic authenticity detection abilities in 6–7-year-olds (Dawel et al., 2015; R. Song et al., 2016). On the other hand, children experienced more difficulty recognizing the authenticity of sad expressions, as reflected in lower recognition accuracy and discrimination indices compared to happy expressions. Specifically, younger children failed to distinguish genuine from posed sad expressions, whereas middle- and upper-grade students’ discrimination indices exceeded chance (>0.5). Previous studies have produced conflicting evidence regarding children’s ability to discriminate genuine from posed sad expressions. While Serrat et al. (2020) claimed that elementary school children could recognize genuine sadness, their study did not directly assess accuracy rates in distinguishing genuine versus posed sad expressions. In contrast, Dawel et al. (2015) concluded that children universally lack this ability throughout elementary school. Our findings provide a clearer characterization of the developmental trajectory of sad authenticity recognition in elementary school children.

This study revealed that children exhibited greater difficulty in recognizing genuine facial expressions compared to posed ones. They demonstrated superior recognition accuracy in identifying happy expressions relative to sad ones. An interactive effect between expression and authenticity was observed during perceptual evaluation. Elementary school children showed enhanced accuracy for both genuine and posed happy expressions, consistent with established findings (Dawel et al., 2015; R. Song et al., 2016). Children’s enhanced ability to discriminate authenticity in happy expressions may stem from the “smiling face recognition advantage”. Previous research has consistently demonstrated that happy faces capture attention faster and are recognized more accurately and quickly than other basic expressions. A related study revealed that smiling faces were the preferred facial expressions in figure drawings created by 6–13-year-old children, suggesting a correlation between artistic depictions of facial expressions and facial expression perception skills (Cannoni et al., 2021). This positivity preference is reflected in our reaction time data: students beyond the lower-grade level recognized happy expressions significantly faster than sad ones. The observed difficulties in recognizing genuine sad expressions may stem from children’s limited understanding of adult sad display rules. Compared to positive emotions like happiness, adults seldom exhibit overt negative emotions such as sadness in children’s presence, resulting in insufficient exposure to adult-style facial cues of genuine sadness. Post-experiment interviews revealed children’s stereotypical belief that sadness should manifest through crying and tears—expressive patterns they primarily observed in peer interactions. In contrast, adult sad expressions are perceived as more restrained, characterized by subtle muscle movements in the Eye-region (Fu, 2022). This self-referential cognitive framework leads children to misinterpret adult-specific facial cues, which reduces genuine sad recognition accuracy. While adult ratings provide standardized authenticity benchmarks, they may not fully capture children’s perceptual weighting of cues. Future studies should employ cross-age rating paradigms to quantify developmental shifts in cue prioritization and to disentangle true perceptual maturation from acquisition of adult-like standards.

Using eye-tracking technology, we systematically examined children’s gaze patterns during facial expression authenticity judgments. Children exhibited the longest fixation durations on the Eye-region, followed by the Midface, and then the Mouth. This pattern likely arises because dynamic brow-eye movements provide essential cues for recognizing authentic happy (Calvo et al., 2012) and sad expressions (Fu, 2022). This gaze pattern aligns with findings in adult populations. Manera et al. (2011) demonstrated that participants exhibited significantly longer fixations on the Eye-region compared to the mouth region when discriminating between spontaneous and posed smiles based on Duchenne markers. The Midface emerges as the second most prioritized facial region, likely related to its geometric centrality, a phenomenon termed the “facial centroid effect” (Gu & Bai, 2014). This central positioning allows observers to efficiently scan between key emotion-signaling regions (eyes and mouth) with minimal visual distances to acquire critical recognition cues.

The present study examined whether the developmental improvements in expression authenticity recognition accuracy were associated with gaze preference patterns across facial regions. Results indicated children’s ability to distinguish genuine from posed facial expressions improves with age, yet their visual attention patterns, consistently prioritizing the Eye-region, remain stable across development. This directly challenges the assumption that older children enhance their accuracy through increased attention to diagnostic cues like eye/brow movements. These findings align with prior work demonstrating no significant correlation between gaze patterns and detection accuracy (Manera et al., 2011; Perron & Roy-Charland, 2013). Furthermore, trial-level analyses showed that fixation duration did not predict judgment accuracy, reinforcing that attentional mechanisms are not the primary driver of developmental improvement. These results suggest the PAM framework, which posits attentional focus as crucial for detecting authenticity, may be better suited to experimental settings using artificially highlighted cues (like exaggerated Duchenne markers). In more naturalistic contexts like ours, where facial cues appear in realistic combinations, the core mechanism underlying children’s improved detection skills likely involves the maturation of socio-cognitive abilities (e.g., Theory of Mind), rather than attention pattern adjustments. Existing research identifies two core ToM competencies essential for detecting posed emotions: appearance-reality distinction (differentiating superficial displays from genuine emotional states) and false belief understanding (recognizing that others’ expressions may misrepresent internal feelings) (Harris et al., 1986). Children demonstrate the ability to distinguish between appearance and reality in objects by age 3 (Flavell et al., 1983), gradually extending this capacity to emotional contexts. While 6-year-olds begin grasping emotional false beliefs, knowing that others’ expressions may not reflect genuine feelings (Devine & Hughes, 2013). Wellman et al. (2001) demonstrated that 3- to 4-year-olds performed at chance levels (50%) on ToM tasks, with accuracy rates increasing systematically until near-mastery by age 8. This developmental trajectory suggests that marked improvements in facial authenticity discrimination between lower and middle grades may reflect accelerated maturation of ToM capacities. Moreover, improvements in children’s authenticity discrimination skills may also be supported through cumulative socio-cultural learning and enriched peer interaction experiences, which refine emotion decoding strategies.

While this study advances understanding of developmental trajectories in children’s ability to differentiate genuine versus posed expressions and their visual attentional patterns across facial regions, several methodological constraints warrant consideration. First, ecological validity may be constrained by laboratory-controlled static expression stimuli lacking social contexts and real-world dynamic cues. Facial authenticity judgments primarily occur in social contexts, where individuals use environmental cues and situational knowledge to interpret expressions (Maringer et al., 2011). Empirical evidence demonstrates that socio-contextual factors systematically modulate the perception of a smile in the face (Krumhuber et al., 2021; Mui et al., 2020). Future research should investigate the impact of contextual or situational information on children’s ability to recognize the authenticity of different facial expressions to improve ecological validity. Second, our study employed a sequential presentation format for facial expressions. Future research could explore alternative presentation methods, such as displaying genuine and posed exemplars of the same emotion side-by-side. This simultaneous presentation may facilitate direct feature comparison, potentially enhancing authenticity discrimination accuracy, particularly for expressions like sadness, where diagnostic cues are subtler. Investigating the differences in performance and eye-movement patterns between sequential and simultaneous presentation formats could provide further insights into whether underlying processes rely more heavily on perceptual feature comparison or conceptual representations of authenticity. Third, while the current study focused specifically on happy and sad expressions, future work should investigate authenticity discrimination across diverse basic emotions (e.g., surprise, disgust, and fear) to establish generalizable developmental trajectories. Critically, such comparative analyses will elucidate whether the observed dissociation between gaze pattern stability and socio-cognitive maturation characterizes a domain-general mechanism or is cued specifically to periocular-dependent expressions. Fourth, building on our finding that children transition from behavioral to neuromuscular cue reliance during socialization, future work should compare gaze patterns during peer versus adult expression decoding and examine neural correlates of child- versus adult-validated cues. This will elucidate how neurobiological perception and socially transmitted standards collectively drive the developmental progression observed in authenticity detection. Finally, cultural factors critically shape both the display and perception of authentic versus posed emotions (Fang et al., 2022). Future cross-cultural comparisons are needed to determine whether the observed developmental trajectory reflects universal mechanisms or culture-specific learning processes.

## 5. Conclusions

The results of the present study indicated that elementary school children exhibited systematic improvements in recognizing both genuine versus posed happy and sad expressions across grade levels, with upper-grade students demonstrating significantly faster reaction times and higher accuracy rates. Notably, this enhanced accuracy was unrelated to shifts in gaze patterns during authenticity judgments of facial expression. Children across all grades consistently prioritized the Eye-region, which provides essential cues for recognizing authentic facial expressions, thereby challenging the perceptual-attentional mechanisms framework. Moreover, elementary school children demonstrated differentiated authenticity discrimination capacities across facial expression types, with significantly higher accuracy in distinguishing genuine versus posed happy expressions compared to sad ones. These findings delineated a clearer developmental trajectory of authenticity recognition of happy and sad expressions among elementary school children.

## Figures and Tables

**Table 1:** Means and standard deviations of recognition accuracy across grades in different conditions.

Expression	Authenticity	Grade		
		Lower	Middle	Upper
Happy	Genuine	0.64 (0.48)	0.80 (0.40)	0.80 (0.40)
	Posed	0.75 (0.44)	0.74 (0.44)	0.82 (0.38)
Sad	Genuine	0.49 (0.50)	0.53 (0.50)	0.55 (0.50)
	Posed	0.73 (0.45)	0.76 (0.43)	0.73 (0.45)

**Table 2:** Means and standard deviations of the discrimination index across grades in different conditions.

Expression	Grade		
	Lower	Middle	Upper
Happy	1.11 (0.96)	1.59 (0.82)	1.94 (0.89)
Sad	0.67 (0.74)	0.80 (0.44)	0.85 (0.60)

**Table 3:** Means and standard deviations of Reaction time (ms) across grades in different conditions.

Expression	Authenticity	Grade		
		Lower	Middle	Upper
Happy	Genuine	1885 (2294)	1417 (1598)	1119 (1027)
	Posed	1957 (1931)	1653 (1671)	1291 (1132)
Sad	Genuine	1880 (1948)	1741 (1506)	1445 (1362)
	Posed	1857 (1738)	1864 (1648)	1386 (1147)

**Table 4:** Means and standard deviations of fixation duration (ms) across grades in different conditions.

Expression	Authenticity	Area of Interest	Grade		
			Lower	Middle	Upper
Happy	Genuine	Eye-region	1717 (848)	1689 (878)	1487 (883)
		Mouth	1158 (811)	1039 (772)	1002 (866)
		Midface	831 (654)	901 (736)	849 (681)
	Posed	Eye-region	1911 (1018)	1969 (911)	1779 (978)
		Mouth	874 (791)	755 (703)	750 (728)
		Midface	888 (743)	861 (651)	869 (730)
Sad	Genuine	Eye-region	2400 (1020)	2134 (940)	2055 (1012)
		Mouth	495 (573)	409 (507)	429 (563)
		Midface	884 (691)	1011 (676)	909 (794)
	Posed	Eye-region	1946 (963)	1814 (959)	1663 (1019)
		Mouth	601 (620)	527 (535)	588 (714)
		Midface	986 (698)	1127 (750)	1024 (783)

**Table 5:** Means and standard deviations of Eye-region fixation duration (ms) across grades in different conditions.

Expression	Authenticity	Detection Response	Grade		
			Lower	Middle	Upper
Happy	Genuine	Correct	1687 (865)	1684 (904)	1428 (875)
		Incorrect	1769 (818)	1709 (779)	1757 (876)
	Posed	Correct	1858 (980)	1987 (888)	1734 (980)
		Incorrect	2074 (1115)	1918 (977)	2003 (945)
Sad	Genuine	Correct	2484 (905)	2141 (941)	2160 (973)
		Incorrect	2321 (1116)	2126 (941)	1875 (1036)
	Posed	Correct	1878 (943)	1812 (907)	1685 (999)
		Incorrect	2115 (995)	1821 (1111)	1602 (1073)

## Data Availability

The original data presented in this study are openly available via the OSF at https://osf.io/h6bas/ (accessed on 30 March 2025).
